# Longitudinal CMR assessment of cardiac global longitudinal strain and hemodynamic forces in a mouse model of heart failure

**DOI:** 10.1007/s10554-022-02631-x

**Published:** 2022-05-21

**Authors:** Mariah R. R. Daal, Gustav J. Strijkers, David J. Hautemann, Aart J. Nederveen, Rob C. I. Wüst, Bram F. Coolen

**Affiliations:** 1 Department of Biomedical Engineering and Physics, Amsterdam University Medical Centers, Amsterdam Cardiovascular Sciences, University of Amsterdam, Meibergdreef 9, 1105 AZ Amsterdam, The Netherlands; 2 Medis Medical Imaging Systems B.V, Leiden, The Netherlands; 3 Department of Radiology and Nuclear Medicine, Amsterdam University Medical Centers, Amsterdam Cardiovascular Sciences, University of Amsterdam, Amsterdam, The Netherlands; 4 Laboratory for Myology, Department of Human Movement Sciences, Faculty of Behavioral and Movement Sciences, Vrije Universiteit Amsterdam, Amsterdam Movement Sciences, Amsterdam, The Netherlands

**Keywords:** Heart failure, HFpEF, MRI, Strain, Hemodynamic forces, Diabetes, Mouse

## Abstract

To longitudinally assess left ventricle (LV) global longitudinal strain (GLS) and hemodynamic forces during the early stages of cardiac dysfunction in a mouse model of heart failure with preserved ejection fraction (HFpEF). Cardiac MRI measurements were performed in control mice (n = 6), and db/db mice (n = 7), whereby animals were scanned four times between the age of 11–15 weeks. After the first scan, the db/db animals received a doxycycline intervention to accelerate progression of HFpEF. Systolic function was evaluated based on a series of prospectively ECG-triggered short-axis CINE images acquired from base to apex. Cardiac GLS and hemodynamic forces values were evaluated based on high frame rate retrospectively gated 2-, 3-, and 4-chamber long-axis CINE images. Ejection fraction (EF) was not different between control and db/db animals, despite that cardiac output, as well as end systolic and end diastolic volume were significantly higher in control animals. Whereas GLS parameters were not significantly different between groups, hemodynamic force root mean square (RMS) values, as well as average hemodynamic forces and the ratio between hemodynamic forces in the inferolateral-anteroseptal and apical–basal direction were lower in db/db mice compared to controls. More importantly, hemodynamic forces parameters showed a significant interaction effect between time and group. Our results indicated that hemodynamic forces parameters were the only functional outcome measure that showed distinct temporal differences between groups. As such, changes in hemodynamic forces reflect early alterations in cardiac function which can be of added value in (pre)clinical research on HFpEF.

## Introduction

Heart failure can be roughly categorized into heart failure with preserved ejection fraction [HFpEF with left ventricular ejection fraction (LVEF) ≥ 50%], heart failure with midrange ejection fraction (HFmrEF with LVEF between 40 and 49%), and heart failure with reduced ejection fraction (HFrEF with LVEF < 40%). Early diagnosis is critical for effective treatment and may differ for each category depending on the underlying etiology [1–3]. Because clear clinical signs or symptoms are often absent in the early phases of the disease, a straightforward diagnosis of HFpEF remains difficult and requires objective evidence of cardiac structural and functional alterations [2, 4]. The heterogeneous pathophysiology of HFpEF includes impaired diastolic filling, stiffening of the myocardium, atrial dysfunction, and pulmonary hypertension [5], which can be related to renal disease[5, 6], type 2 diabetes mellitus [5, 7] and hypertension [5, 8].

Non-invasive methods to measure subtle changes in myocardial structure and function underlying HFpEF are therefore highly desired both in clinical as well as in preclinical research to monitor disease progression in HFpEF and to study the effects of medical treatment or interventions [9–11].

Cardiac magnetic resonance (CMR) imaging has emerged as a versatile translational imaging modality for the characterization of HFpEF, offering a variety of cardiac structural and functional outcome measures with comparable protocols for humans and small animals [12]. Recent developments in CMR acquisition and post-processing methods such as feature tracking and advanced flow modeling have even created new opportunities to quantitatively assess cardiac function in HFpEF beyond LVEF. Specifically, clinical studies have already shown feature tracking to have good reproducibility for the assessment of cardiac strain [13–15], which has shown diagnostic and prognostic potential in HFpEF patients [16–18]. Lower absolute values for global longitudinal strain (GLS) were observed despite preserved LVEF values, potentially due to compensatory changes in global circumferential strain (GCS), wall thickness, or diameter of the left ventricle [19]. Intracardiac hemodynamic forces have also shown potential to assess cardiac function beyond LVEF, and can be estimated in humans with a mathematical model using feature tracking on conventional LV 2-, 3- and 4-chamber CINE MR images [20]. The hemodynamic force represents the force exchange between ventricular blood and surrounding myocardium and is a global measure of the interventricular pressure gradient integrated over the LV volume [20]. Alterations in hemodynamic forces over the cardiac cycle indicate an alteration in blood-tissue interaction, possibly both a cause and effect of the progression of structural remodeling [21]. Lapinskas et al*.* recently demonstrated the use of hemodynamic forces to distinguish patients with normal LVEF, HFpEF, HFmEF and HFrEF [22]. Lower hemodynamic forces in patients with HFpEF compared to healthy volunteers were observed, without a significant difference in LVEF or GLS. As such, hemodynamic forces can be an important marker for cardiac functional changes in the early phases of myocardial dysfunction in HFpEF [22–24]. Recently, our group demonstrated the feasibility of calculating hemodynamic forces in mice using conventional LV 2-,3- and 4-chamber CINE CMR images in combination with clinically validated software that implements this mathematical model [25]. However, the feasibility to use this parameter for preclinical studies in HFpEF has never been assessed before.

In order to study possible distinct temporal behavior of several cardiac functional parameters during early development of heart failure, we therefore applied a comprehensive preclinical CMR protocol for longitudinal characterization of cardiac LV dysfunction. Specifically, we compared changes in LV functional parameters between healthy control mice and a diabetic mouse model (db/db mice) treated with the antibiotic doxycycline. The db/db mice are a known model for diastolic dysfunction with preserved ejection fraction thus mimicking aspects of human HFpEF [26]. Recently we observed that administration of doxycycline exacerbates development of diastolic dysfunction in db/db mice accelerating the progression of HFpEF [27], due to metabolic and mitochondrial dysfunction [27, 28]. As such, we deemed that this model provided us with a feasible intervention that accelerated HFpEF disease progression, better than for instance a myocardial infarction that leads to obvious, rapid and widespread alterations in the LVEF and hence GLS and/or hemodynamic forces. For our experimental setup, we hypothesized that db/db mice would exhibit early changes in GLS and/or hemodynamic force parameters, with distinct temporal behavior from healthy controls.

## Materials and methods

### Animals

**Fig. 1:**
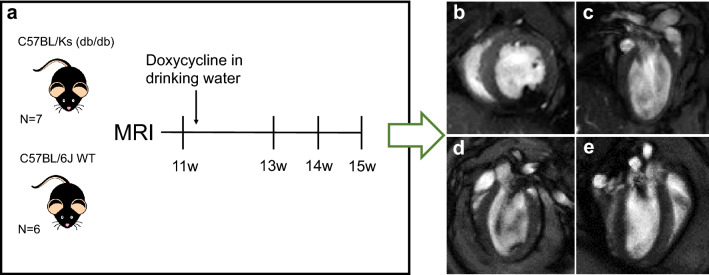
Study design. **a** Overview of study timeline and start of doxycycline treatment for db/db animals. **b**–**e** Representative end-diastolic static frames from CINE movies in **b** midventricular short-axis, **c** long-axis 2-chamber, **d** 3-chamber, and **e** 4-chamber orientations

All in vivo experiments were conducted in compliance with the Dutch government guidelines and approved by the Animal Welfare Committee of the Amsterdam Medical Center, Amsterdam. Eight C57BL/KsOlaHsd-Lepr (db/db) male mice and six C57BL/6 (control) male mice (purchased from Envigo, Horst, Netherlands) were included in the study. Animals were housed in pairs in IVC cages in a facility with a 12 h day-night cycle, 50% humidity, and at 22 °C. All animals could eat and drink ad libitum and received standard chow. Figure 1a provides a schematic overview of the study design. Cardiovascular magnetic resonance (CMR) measurements at four time points over a period of 4 weeks at the age of 11, 13, 14, and 15 weeks to follow temporal alterations in cardiac function. After the first MRI scan, the db/db animals received 5 g/L doxycycline 5% sucrose in their drinking water.

### Cardiovascular MRI

CMR measurements at each time point were performed with a 7-Tesla MRI (MR Solutions Ltd., Guildford, UK) using a 38-mm-diameter mouse birdcage coil (MR Solutions Ltd., Guildford, UK), as described in our previous paper [25]. In short, animal anesthesia was induced by inhalation of 4% isoflurane and maintained at 1–2% isoflurane mixed with 0.2 mL/min Air and 0.2 mL/min O_2_ during experiments. Eye ointment was applied to avoid drying out of the cornea. Animals were kept warm with a hot air heating bed (Minerve, Esternay, France) to maintain an internal body temperature of 36–37 °C. Temperature was measured with a rectal probe (Opsens Solutions’ OTP-M fiber optic temperature sensor, Québec, Canada) and monitored throughout the experiment (Opsens AccuSens, Québec, Canada). A respiratory balloon was placed on the abdomen directly under the sternum and 2 ECG leads were inserted subcutaneously on the left and right side of the chest for respiration and ECG monitoring using the PC-SAM software (Small Animal Instruments, Inc., Stony Brook, NY, USA).

For systolic function measurements, a series of prospective ECG- and respiratory triggered short-axis CINE-movies were acquired covering the heart from apex to base with a FLASH sequence using the following parameters: TR = 7 ms, TE = 2.5 ms, flip angle = 20°, FOV = 35 × 35 mm^2^, acquisition matrix = 192 × 192, slice thickness = 1 mm, number of slices = 7–9 (depending on heart size), number of cardiac frames = 12–17 (depending on heart rate), number of averages = 5, scan time = 20 min. End-systolic (EDV) and end-diastolic volumes (ESV), stroke volume (SV), ejection fraction (EF), and cardiac output (CO) were determined by delineating endomyocardial borders in the end-systolic and end-diastolic frames in all slices, using MEDIS suite MR software (Medis Medical Imaging Systems BV, Leiden, The Netherlands).

To assess global longitudinal strain and hemodynamic forces, single-slice retrospectively-gated CINE-images were acquired in 2-, 3-, and 4-chamber orientations (Fig. 1c–e) using a FLASH sequence with a slice-excitation based navigator for detecting respiratory and cardiac motion. CINEs were acquired with the following parameters: TR = 8 ms, TE = 2.4 ms, flip angle = 15°, field of view = 30 × 30 mm^2^, acquisition matrix size = 192 × 192, slice thickness = 1 mm, number of k-space repetitions = 400, scan time = 13 min. Retrospective triggering allows for obtaining a higher temporal resolution than the specific TR value used. The acquired data was therefore retrospectively reconstructed into 32 cardiac time frames based on custom-made and open-source software [25], which uses Matlab reconstruction algorithms as well as compressed sensing code from the Berkeley Advanced Reconstruction Toolbox (BART). All reconstructed CINE-images were visually evaluated to ensure the CINE displayed a smooth contractile motion of the myocardium similar to the prospectively triggered CINE-movies. LV endocardial walls in the 2-, 3-, and 4-chamber view CINE-images were delineated using the QMass plugin in MEDIS software (MEDIS, Leiden, the Netherlands). From these delineations, the QStrain plugin was used to calculate the endomyocardial global longitudinal strain (endoGLS) throughout the duration of the cardiac cycle and the peak endoGLS, as well as the root means square (RMS) and average LV hemodynamic forces in the apical-basal direction throughout the duration of the cardiac cycle, based on previously described mathematical equations [20, 29, 30].

### Statistics

All values are reported as mean ± SD. Statistical analysis was performed using Graphpad Prism 9.1.2. For one specific case where the acquired data could not be delineated due to poor image quality, mean substitution was applied. Effects of both time and group (control vs. db/db) and their interaction were analyzed using repeated measures ANOVA. If no interaction was found, the analysis was repeated including only main effects, followed by a Tukey’s test for post-hoc analysis with statistical significance set at 0.05.

## Results

Data of 7 db/db animals was included in the analysis as one animal needed to be euthanized due to unexpected health complications prior to the second scan. All functional parameters investigated in this study are summarized in Table 1 as well as described in more detail in the section below.

**Table 1:** Left ventricular functional parameters of db/db and control animals

	db/db	control	p value
LV ESV (µL)	11 ± 3	16 ± 3	0.01
LV EDV (µL)	45 ± 5	56 ± 8	< 0.01
EF (%)	74 ± 3	71 ± 3	0.08
CO (mL/min)	20 ± 2	28 ± 4	< 0.01
EndoGLS (%)	− 23 ± 3	− 22 ± 3	0.10
HDF RMS (%)	90 ± 24	133 ± 41	0.01
HDF average (%)	38 ± 12	67 ± 18	< 0.01
HDF ratio (%)	− 3 ± 2	− 1 ± 1	< 0.01

Data are expressed as mean ± SD

*LV *left ventricle, *ESV *end-systolic volume, *EDV* end-diastolic volume, *EF *ejection fraction, *CO* cardiac output, *EndoGLS *endomyocardial global longitudinal strain, *HDF *hemodynamic force, *RMS* root mean square

### LV systolic function

**Fig. 2:**
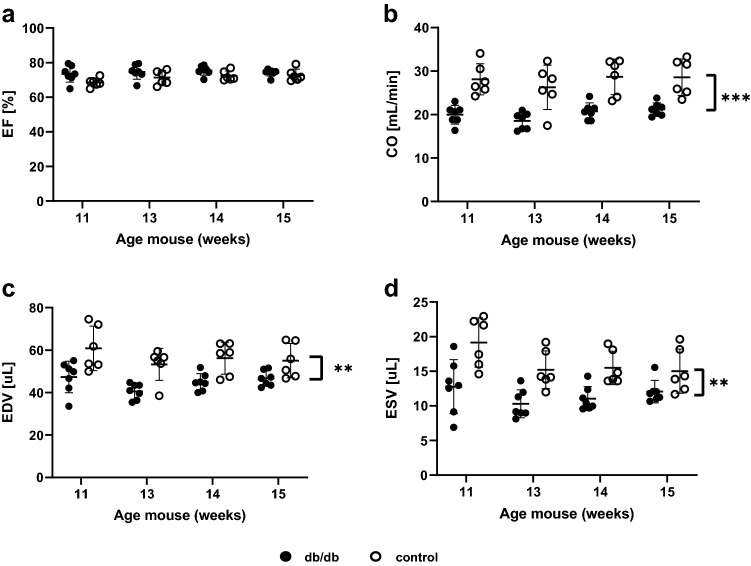
Left ventricular systolic function analysis over time for control and db/db animals. **a** Ejection fraction **b** Cardiac output, **c** End-diastolic volume and **d** End-systolic volume. All measurements in week 1 are baseline measurements, prior to treating the db/db animals with doxycycline. **p ≤ 0.01, ***p ≤ 0.001 for group

Systolic function was quantified from the series of prospectively triggered multi-slice short-axis CINE-images. Fig. 2a displays results for the ejection fraction, which tended to be slightly higher in db/db animals (74 ± 3%) compared to controls (71 ± 3%; p = 0.08). Ejection fraction in both groups remained above 50% during the follow-up period, thus indicating preserved ejection fraction of the doxycycline-treated db/db mice. No significant interaction effect between time and group was found, and only a small increase in ejection fraction was observed in the follow-up period in both groups (p = 0.02 between time point 1 and 3), likely due to growth-induced reductions in cardiac volumes.

The results for cardiac output (CO), and end-diastolic(EDV) and end-systolic volume(ESV) are presented in Fig. 2b–d, respectively. No significant interaction effect between group and time was found for CO, EDV, and ESV. Mean CO was significantly higher in control (28 ± 4 mL/min) compared to db/db (20 ± 2 mL/min) animals (p < 0.01), mainly due to differences in volume, rather than heart rate during the scan. Also, EDV was significantly higher in controls (56 ± 8 µL) compared to db/db animals ( 45 ± 5 µL; p < 0.01). Similarly, ESV was significantly higher in controls (16 ± 3 µL) compared to db/db animals ( 11 ± 3 µL; p = 0.01). Over the course of 4 weeks, both end-diastolic and end-systolic volumes decreased (both p ≤ 0.01).

### Global longitudinal strain

**Fig. 3:**
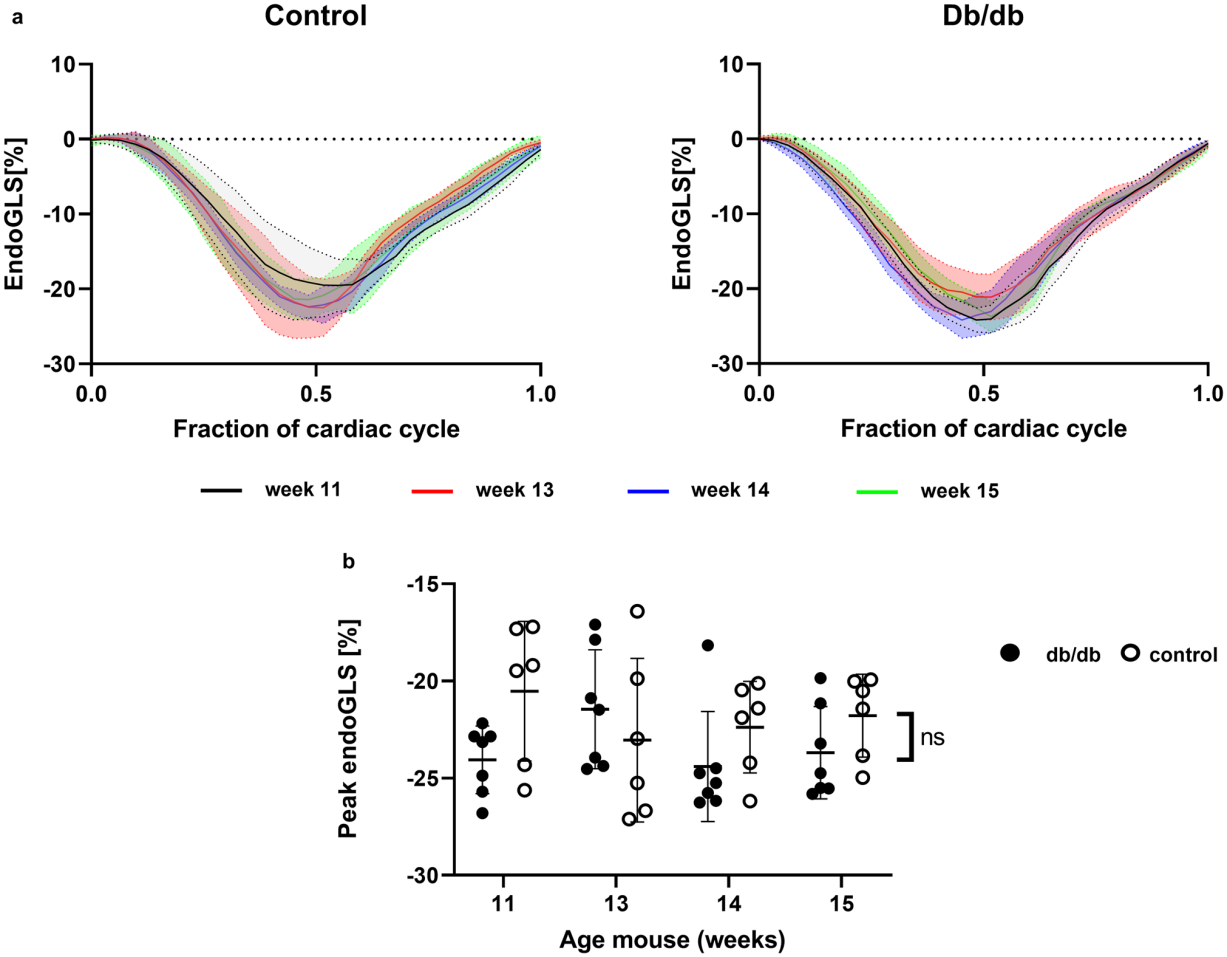
EndoGLS analysis. **a** EndoGLS over time in control animals **b** and db/db animals with mean represented as solid line and SD as dashed lines. **c** Peak Endo GLS values

The endoGLS quantifies the longitudinal shortening of the heart in each cardiac phase with respect to its initial length in end-diastole as a negative percentage [31]. Fig. 3a shows the endoGLS as function of the cardiac phase and in Fig. 3b, peak endoGLS values for both animal groups and four time points are presented. We observed slightly lower peak endoGLS values for db/db mice (− 23 ± 3%) as compared to control mice (− 22 ± 3%), but this was only trend significant (p = 0.10). No significant time (p = 0.68) or interaction effect (p = 0.15) was found.

### Hemodynamic forces

**Fig. 4:**
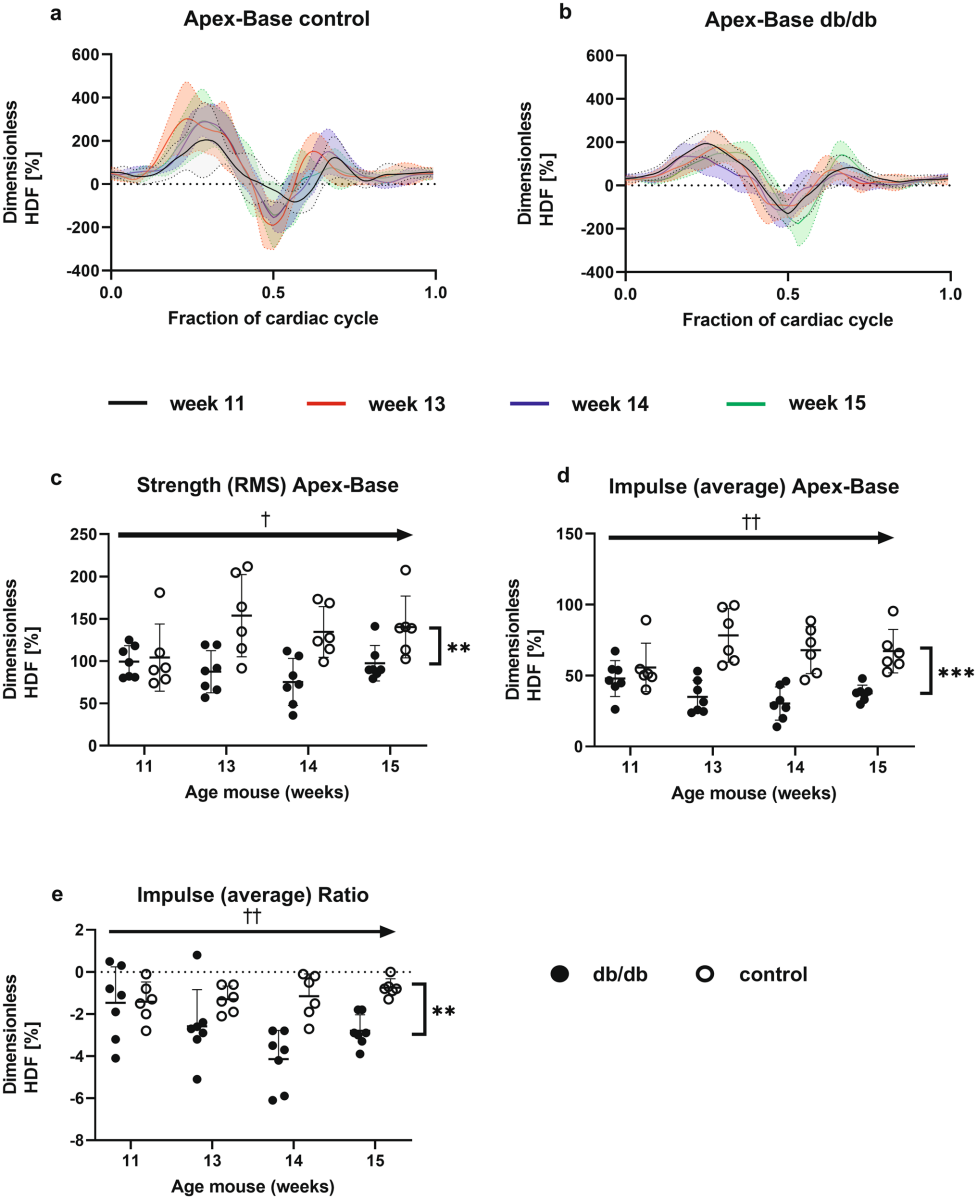
Hemodynamic force profile for **a** control and **b** db/db animals over one cardiac cycle. **c** The root mean square HDF in the apical-basal direction for both groups. **d** The average of the HDF in the apical-basal direction for both groups. **e** The ratio between root mean square of the HDF in iLat-aSep and apical-basal direction. **p < 0.01, ***p < 0.001 for group; † p < 0.05, †† p < 0.01 for interaction

Figure 4a and 4b show hemodynamic forces in apical-basal direction throughout the cardiac cycle for controls and db/db mice, respectively. Hemodynamic forces increase during the systolic ejection phase in the direction of the aorta (denoted as positive values), followed by a downward force towards the apex during transition from systole to diastole noted as a negative value. Hemodynamic forces increase the early diastolic filling deceleration (during the E wave), and again during the late diastolic filling phase (the A wave). Whereas, the overall shapes of the hemodynamic forces profiles were similar between control and db/db mice, the systolic ejection force peaks were consistently lower for db/db mice.

We determined the root mean square of the hemodynamic forces (RMS-HDF) over the whole cardiac cycle as a measure of the overall force amplitude, the average hemodynamic forces which incorporates force direction (positive/negative), and the ratio of average hemodynamic forces in inferolateral (iLat)-anteroseptal (aSep) and apical-basal directions (Fig. 4c–e). For all hemodynamic force parameters, a significant interaction effect between group and time was found (RMS-HDF: p = 0.02, average HDF: p < 0.01, HDF ratio: p < 0.01). Indeed, Fig. 4 c-e shows hemodynamic forces parameters in control mice followed an upward trend (RMS-HDF) or remained stable (average and ratio), whereas in db/db mice, hemodynamic forces in the apical-basal direction are reduced over time. The mean value of the hemodynamic forces parameters are significantly higher for control animals (HDF-RMS 133 ± 41%, HDF average 67 ± 18%, HDF ratio -1 ± 1%) compared too db/db animals (HDF-RMS 90 ± 24%, HDF average 38 ± 12, HDF ratio -3 ± 2%; p ≤ 0.01). This difference between groups is most likely due to the decrease in systolic force peak in the db/db animals compared to control animals as visible in Fig. 4a and 4b.

## Discussion

In this study we aimed to assess whether differences in global longitudinal strain and hemodynamic force parameters occur in an animal model of the early phases of heart failure with preserved ejection fraction (HFpEF) compared to healthy mice, and whether these parameters are a sensitive new imaging parameters to assess alterations in cardiac function during the progression of HFpEF. For this purpose, we used a group of db/db mice treated with doxycycline to exacerbate early development of heart failure and compared these to untreated healthy controls, as two extremes of test groups to detect early alterations in cardiac function. Our results show a significant reduction in hemodynamic force values in db/db animals over time compared to control animals, while there was no significant difference detected for EF and GLS. This suggests that hemodynamic force values can be used as an early biomarker of cardiac dysfunction and provide unique information when studying early HFpEF development or developing treatment strategies for prevention of heart failure.

### Ejection fraction and cardiac strain in HFpEF

Ejection fraction did not differ between groups, however CO, ESV, and EDV were smaller for the db/db mice as compared to controls. These results are consistent with observations in HFpEF patients [2, 32] and previous work with db/db mice [27]. EF did significantly increase over time; however this was not group dependent. Our results show that the EDV and ESV of db/db animals are both reduced with a similar factor compared to the control animals. Furthermore, EDV and ESV changes over time are similar between db/db and control mice, which could explain why there is no significant difference in EF between the two groups, but there is a significant effect of time on the EF for both groups. This also explains the significant difference in CO between the two groups, with no significant change over time.

Mean endocardial global longitudinal strain (endoGLS) values were determined from 2-,3- and 4-chamber view CINE images. This approach has the distinct advantage that it relies on regular CINE acquisitions, in contrast to for instance MRI tagging or DENSE techniques, which require specific pulse sequences and dedicated processing pipelines [33]. Mean endoGLS was similar for control and db/db mice and did not change during the 4-week study period. Observed endoGLS values of the mouse hearts were also comparable to mean values found in humans [13]. A full analysis of myocardial strain would include cardiac deformations in different directions, i.e. longitudinally (shortening from base to apex), circumferentially (circular perimeter shortening) and radially (wall thickening and thinning) [17]. Alterations in these strain values are indicative for changes in the contractile function of the myofibers and possibly fiber damage [16]. Since GLS in particular has been shown to provide a good prognosis for many heart failure-related outcomes independent of EF [34], we decided to focus on this parameter for this study. Previous studies in mouse models of DCM [35], sepsis [36], obesity [37] have shown significant changes in GLS prior to changes in EF. However, we did not find a significant main effect of time for GLS in either of the groups. The time it takes for strain values to change significantly is different for each model, most likely because the difference in progression towards heart failure between the different cardiovascular diseases.

### Hemodynamic forces as a new imaging parameter

Aside from assessment of the GLS, the delineation of the 3 orthogonal views can also be used to assess the hemodynamic force parameters, both in humans [20] and in mice [25]. Until recently, the calculation of hemodynamic forces required cardiac 4D-flow measurements, which are difficult to perform in the small and fast-beating mouse heart and not routinely available on preclinical MRI scanners [38]. To our knowledge, our study is the first to show alteration of hemodynamic force parameters derived from CINE-images in a mouse model of HFpEF. This study supports earlier findings of hemodynamic forces changes prior to functional parameters, including EF, and deformation parameter, such as GLS. More specifically, systolic force peaks were lower for db/db mice, in line with changes in hemodynamic forces that were observed in HFpEF patients [22]. Overall, these results show an altered temporal behavior of hemodynamic force parameters in db/db mice as compared to controls and provide additional support to the previously observed negative effects of the antibiotic doxycycline on cardiac function in this mouse model of diabetes [27]. In healthy subjects, the hemodynamic forces are aligned with the long axis of the LV and in the event of heart failure the hemodynamic forces in iLat-aSep direction increase thus the absolute value of the hemodynamic force ratio between iLat-aSep and apical-basal direction is higher for subjects with heart failure [23]. Particularly interesting is the hemodynamic force ratio between iLat-aSep and apical-basal directions which strongly differed between groups. A shift of the force direction from apical-basal towards iLat-aSep was observed in the db/db mice whereas this force direction remained unchanged in the control group. It was proposed that in the event of global adverse remodeling of the LV, this ratio becomes (negative) larger, as the efficiency of the heart to propel blood in the apical-basal direction becomes less and is accompanied by an increase in the iLat-aSep forces [39].

### Study limitations

The current study has some limitations related to different aspects of the overall design. First, we did not investigate the influence of using a different number of orthogonal views on the resulting GLS and HDF parameters. As there are currently no standardized protocols for CMR feature tracking acquisitions and post-processing [40–42], we chose to use all 3 orthogonal views, which we believe most accurately reflects the total myocardial deformation. However, a drawback of this approach, besides additional scan time, might be that the resulting volume-time curves could suffer from temporal blurring when slight heart rate variations occur between scans, even when using retrospective triggering.

Besides LV functional parameters, literature has shown that also other readouts, such as native and post-contrast T1 mapping, late-gadolinium enhance (LGE) imaging and first-pass perfusion measurements can be of value for assessing the HFpEF [43], which we did not include in our current study. While these parameters have shown to have prognostic value in terms of long-term clinical outcome, in our study we chose to primarily focus on observing possible distinct temporal behavior of novel LV functional parameters, i.e. strain and hemodynamic forces, which are thought to change during early stages of HFpEF. Additionally, for the current protocol including planning of all cardiac orientations, animals were anesthetized between 1.5 h and 2 h. Whereas additional T1 mapping and/or perfusion measurements could have added value to the existing functional read-outs, addition of such scans would also have further increased the length of the protocol and thereby the length of anesthesia, which we considered undesirable in combination with repeated measurements over a time course of 4 weeks.

Finally, concerning our treatment protocol, the doxycycline was dissolved in the drinking water, and animals were housed in pairs, so it was not possible to monitor the exact amount of water each animal consumed. This might have affected the actual dose of doxycycline for each animal.

## Conclusions

Our results indicated that during the early stages of HFpEF, hemodynamic force parameters were the only functional parameters to show a distinct temporal behaviour as compared to age matched healthy control animals. While other functional parameters, such as GLS and EF might also significantly change over longer periods of time as HF progresses, our results suggest alterations in hemodynamic forces provide an unique marker of cardiac dysfunction and would therefore be of added value in preclinical research on early development and treatment strategies for heart failure.

## Data Availability

Open source reconstruction software “Retrospective” available upon request.
